# Fragile skin may benefit from decoration

**DOI:** 10.1016/j.omta.2026.201827

**Published:** 2026-08-14

**Authors:** Tero A.H. Järvinen, Yanling Liao

**Affiliations:** 1 Faculty of Medicine and Health Technology, Tampere University & Tampere University Hospital, Tampere, Finland; 2 Department of Pediatrics, New York Medical College, Valhalla, NY, USA

## Main text

**Figure 1:**
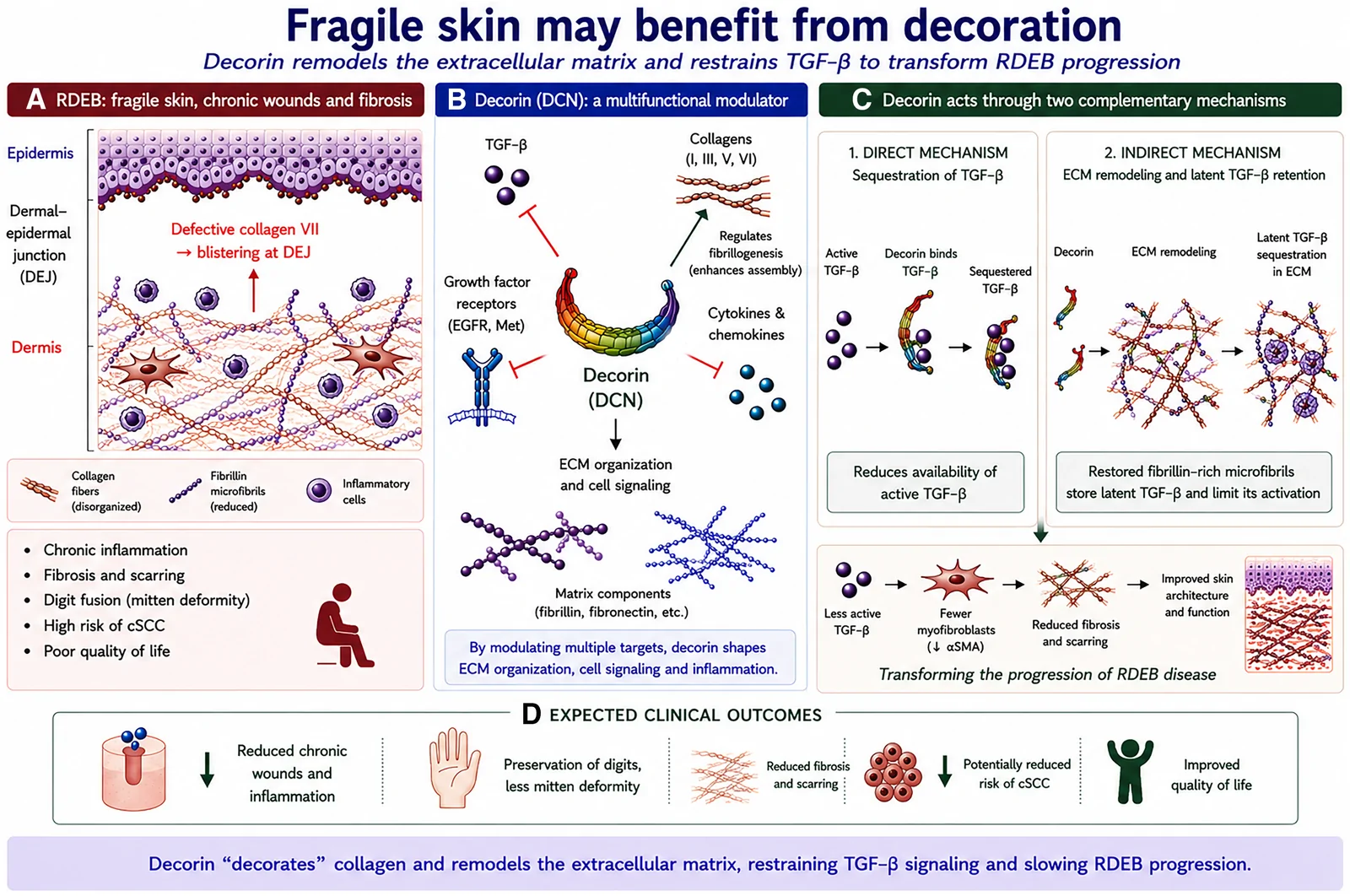
Fragile skin may benefit from decoration (A) RDEB is a severe hereditary blistering disorder characterized by fragility of the skin due to mutations in the *COL7A1* gene, which encodes type VII collagen, the principal component of anchoring fibrils.^1^^,^^2^ Loss or dysfunction of type VII collagen leads to separation at the dermal-epidermal junction, resulting in chronic wounds, scarring, and progressive multisystem complications. (B) DCN is a natural proteoglycan that modulates key signaling pathways by acting as a natural antagonist of TGF-β and several receptor tyrosine kinases. It also regulates collagen fibrillogenesis. (C) Gretzmeier et al.^3^ demonstrate that recombinant human DCN core protein inhibits fibrosis by sequestering TGF-β, increasing fibrillin-1 expression and improving microfibrillar organization in the mice RDEB skin. Among clinically relevant endpoints, DCN treatment improved survival, increased weight gain, reduced skin fibrosis, and inhibited the progression of digit loss in the paws of the RDEB mice.^3^ (D) These preclinical findings support the potential future translation of systemic DCN therapy to treat RDEB patients, with expected clinical benefits including reduced inflammation and fibrosis, preservation of digits and a potentially reduced risk of cSSC and improved quality of life.

Recessive dystrophic epidermolysis bullosa (RDEB) is a severe hereditary blistering disorder characterized by fragility of the skin and mucous membranes due to mutations in the *COL7A1* gene, which encodes type VII collagen, the principal component of anchoring fibrils^1^^,^^2^ (Figure 1A). Loss or dysfunction of type VII collagen leads to separation at the dermal-epidermal junction, resulting in chronic wounds, scarring, and progressive multisystem complications. RDEB is associated with substantial morbidity, including strictures in esophagus, pseudosyndactyly (fusion of fingers), nutritional impairment, and an exceptionally high lifetime risk of developing aggressive cutaneous squamous cell carcinoma (cSCC)^1^^,^^2^ (Figure 1A).

Despite recent therapeutic breakthroughs, including regulatory approvals for local COL7A1 gene and cell therapies for individual skin wounds,^1^^,^^2^ significant challenges remain. After all, RDEB is a systemic disease that affects not only the entire skin but also extracutaneous organs. This underscores the urgent need for systemic therapies. An emerging alternative approach—systemic administration of recombinant decorin (DCN)—is presented in a recent issue of *Molecular Therapy Advances*.^3^

DCN is a small leucine-rich proteoglycan (SLRP) with a single dermatan or chondroitin sulfate glycosaminoglycan (GAG) chain attached to its core protein^3^ (Figure 1B). Originally named for its ability to “decorate” collagen fibers,^4^ DCN regulates collagen fibrillogenesis in the extracellular matrix (ECM) of different tissues, skin among them^3^^,^^4^ (Figure 1B). Beyond its structural role in the ECM, DCN modulates key signaling pathways by acting as a natural antagonist of transforming growth factor-β (TGF-β) and several receptor tyrosine kinases, including epidermal growth factor receptor (EGFR) and Met^4^ (Figures 1B and 1C)). Through these interactions, DCN exerts antifibrotic and antitumorigenic effects.^4^ Owing to its biomechanical role in the skin and its antifibrotic, anti-inflammatory, and tumor-suppressive properties, DCN has emerged as a therapeutic candidate to mitigate the consequences of COL7A1 deficiency in RDEB patients.^3^

Growing body of recent research supports this concept. Initial evidence demonstrated that DCN acts as a phenotypic modifier in monozygotic RDEB twins with identical genetic backgrounds but differing disease severity.^5^ Whole-genome gene expression analysis identified DCN as differentially expressed gene between the monozygotic twins, with significantly higher levels in the less affected twin than in the sibling with a more severe phenotype.^5^ Odorisio et al. also demonstrated that DCN proteoglycan could inhibit the fibrotic processes in fibroblasts derived from sibling with more severe RDEB.^5^ A similar genetic study identified another SLRP, PRELP or prolargin, as a phenotypic modifier, showing higher expression in the twin with milder disease.^6^ Although much is not known about PRELP, it shares structural homology with DCN and also exhibits its key biological functions, namely inhibition of TGF-β signaling and binding to fibrillar collagens.^6^ More recently, a comprehensive analysis of ECM proteins from cell lines derived from human patients with DEB demonstrated that DCN expression decreases with increasing disease severity.^7^

Further support for DCN as a disease-modifying therapeutic in RDEB comes from preclinical studies. First, it was demonstrated that systemically administered recombinant DCN targeted to tissues undergoing repair, could significantly inhibit scar formation in skin wounds.^4^ Then, upregulation of DCN expression was identified as one mechanism underlying the therapeutic effects of cord-blood-derived stem cells in *Col7a1*-deficient (RDEB) mice.^8^ Local DCN gene therapy using lentiviral vectors attenuated fibrosis in an RDEB mouse model.^9^ Moreover, systemic administration of a recombinant fusion protein combining DCN with a skin-homing and tissue-penetrating peptide (tCRK), referred to as DCN-tCRK, significantly improved survival in a lethal RDEB mouse model.^10^ This multifunctional molecule also suppressed inflammation and fibrosis in the skin.^10^

In a recent issue of *Molecular Therapy Advances*, Gretzmeier et al. bring DCN closer to clinical application in RDEB.^3^ The authors generated a recombinant “clinical-grade” human DCN (called rhDCN) core protein from proteoglycan by mutating the GAG attachment site. Repeated systemic administration of rhDCN in murine model of RDEB was well tolerated and therapeutically effective.^3^ The rhDCN accumulated in the RDEB-ravaged skin. Treatment improved survival, increased weight gain, reduced skin fibrosis, and inhibited the progression of digit loss in the paws of the RDEB mice^3^ (Figures 1C and 1D). Taken together, their study confirms the previous study^10^ that an antifibrotic and anti-inflammatory molecule could have clinically meaningful benefits in the RDEB condition without addressing biomechanical defect caused by COL7A1 deficiency. In larger context, these results confirm the previous experimental treatment results on skin wound healing model^4^ and on lethal RDEB model^10^ that human DCN derived from ECM can be converted to systemically administered therapeutic that has substantial biological activity in severe disease models.

Beyond demonstrating therapeutic efficacy, the study also provides important mechanistic insights into how DCN may modulate fibrosis in RDEB.^3^ DCN has traditionally been viewed as a TGF-β antagonist through sequestration of the growth factor by its core protein. However, Gretzmeier et al. observed increased fibrillin-1 expression and improved microfibrillar organization within the dermal ECM following the rhDCN treatment^3^ (Figure 1C). Because fibrillin-rich microfibrils serve as an important extracellular reservoir for latent TGF-β complexes through interactions with latent TGF-β-binding proteins, these findings raise the possibility that DCN may influence TGF-β bioavailability not only through direct ligand sequestration, but also through modulation of the ECM architecture^3^ (Figure 1C). Interestingly, short-term rhDCN exposure produced little inhibition on TGF-β signaling, whereas prolonged rhDCN treatment resulted in marked suppression of TGF-β signaling and myofibroblast transformation in the RDEB model.^3^ This temporal activity pattern suggests that remodeling of the ECM (and subsequent TGF-β bioavailability) may contribute to DCN’s antifibrotic activity.

The study by Gretzmeier et al. may have also shed more light on RDEB biology as they identified the altered collagen crosslinking in murine RDEB skin.^3^ In contrast to the accumulation of mature, highly cross-linked collagen seen in many fibrotic disorders, murine RDEB skin exhibited reduced collagen cross-linking.^3^ These findings indicate that despite substantial fibrosis developing in murine RDEB skin, the skin ECM may remain in a relatively immature and dynamically remodeled state in RDEB. Such matrix plasticity may provide a biological explanation to why the rhDCN treatment was able to modify skin ECM and delay progression of toe loss in the paws of RDEB mice^3^ (Figure 1D). The ECM plasticity may also expand the therapeutic opportunity for antifibrotics such as DCN in patients with RDEB.

In summary, the work by Gretzmeier et al.^3^ represents a significant step toward translating the long-recognized biological potential of DCN into a clinically relevant therapy for RDEB. By demonstrating that systemically administered, clinical-grade rhDCN is not only well tolerated but also capable of reducing fibrosis and mitigating hallmark disease manifestations in experimental RDEB disease model, such as digit loss in paws (mitten deformity)^3^ (Figure 1D), the study provides compelling evidence to address the critical unmet need in RDEB, i.e., systemic therapies, with DCN in the future clinical trials. While challenges remain—particularly regarding validation in human trials—the study provides experimental proof-of-concept evidence that severe fragile skin disease such as RDEB may benefit from some “decoration” in its ECM.

## Acknowledgments

The authors thank Drs. Eva Engvall and Erkki Ruoslahti (Sanford Burnham Prebys Medical Discovery Institute, La Jolla, CA, USA) for their insightful comments on the editorial. This work was supported by grants from Jane and Aatos Erkko Foundation, Sigrid Juselius Foundation, state funding for university-level health research through Tampere University Hospital, Wellbeing Services County of Pirkanmaa (grant T63764) (all to T.A.H.J.).

## Declaration of interests

T.A.H.J. and Y.L. report a patent on DCN-tCRK that is licensed to Norgine. Norgine has funded the work at their respective laboratories. T.A.H.J. is a consultant to Norgine with a yearly financial benefit exceeding $10,000.
